# Clinical Spectrum and Outcomes of Melioidosis: An Eight-Year Study

**DOI:** 10.7759/cureus.102413

**Published:** 2026-01-27

**Authors:** Chandni Radhakrishnan, Sasidharan Puthenpurakkal Kunhumon, Thulaseedharan N K, Udayabhaskaran V, Remadevi S, Ajitha BK, Dhananjayan Dhanasooraj

**Affiliations:** 1 Internal Medicine, Government Medical College, Kozhikode, Kozhikode, IND; 2 Internal Medicine, KMCT Medical College, Kozhikode, IND; 3 Microbiology, Government Medical College, Kozhikode, Kozhikode, IND; 4 Statistics, Government Medical College, Kozhikode, Kozhikode, IND; 5 Multidisciplinary Research Unit, Government Medical College, Kozhikode, Kozhikode, IND

**Keywords:** abscess, alcoholism, burkholderia pseudomallei, diabetes mellitus, fever of unknown origin, gram-negative bacteremia, melioidosis, risk factors, tuberculosis, whitmore's disease

## Abstract

Objective

This study aimed to describe the clinical spectrum, demographic characteristics, risk factors, and treatment outcomes of patients with culture-proven melioidosis at a tertiary care center in South India.

Materials and methods

We conducted an eight-year retrospective study from September 2004 to August 2012. This study included all inpatients with clinical samples culture-positive for *Burkholderia pseudomallei*. Data on demographics, presentation, comorbidities, management, and outcomes were collected from medical records. All patients received a standardized antibiotic regimen.

Results

Among 34 culture-proven patients, melioidosis showed a strong male predominance (28, 82.4%), with a mean age of 43 ± 14.8 years. Diabetes mellitus (DM) was the most common comorbidity (24, 70.6%). Fever was the most common symptom (30, 88.2%), with musculoskeletal infection (12, 35.3%), particularly septic arthritis (11, 32.4%), as the most frequent focal manifestation. Bacteremia was present in 19 (55.9%) of the cases. The in-hospital mortality rate was 29.4% (10), with trends suggesting higher mortality among older age groups and those with diabetes and jaundice.

Conclusion

Melioidosis presents as a severe, systemic infection with significant mortality in this region of India. A high index of suspicion is essential for early diagnosis, especially in patients with DM, fever, and abscesses. The disease often mimics tuberculosis, which complicates diagnosis. The recommended antibiotic regimen was effective for most patients, highlighting the importance of timely and appropriate antimicrobial therapy to improve outcomes. Increased physician awareness and prompt laboratory support are crucial for early recognition and management.

## Introduction

Melioidosis, a life-threatening infection, is caused by *Burkholderia pseudomallei* (*B. pseudomallei*), a gram-negative environmental bacterium characterized by clinical diversity and diagnostic and therapeutic challenges [1,2]. The global impact of melioidosis is increasing, with cases reported in many areas outside the tropics and northern Australia. Therefore, early detection is essential for effective management of this disease [1].

This saprophytic organism is common in endemic areas, notably Southeast Asia and northern Australia. Often called "the great mimicker," melioidosis exhibits diverse clinical manifestations that can resemble various infections, making diagnosis challenging [3-5]. The present article discusses cases observed over eight years in a tertiary care center in Kerala, South India.

Diabetes mellitus (DM) is a known risk factor that weakens immune responses, increasing vulnerability to infections such as melioidosis [6]. *Burkholderia pseudomallei*, an opportunistic pathogen, can cause severe clinical symptoms in immunocompromised patients. Specifically, the altered immune responses in individuals with DM can delay the mobilization and activation of immune cells against the bacterium, potentially aiding pathogen survival [6]. Moreover, melioidosis is increasingly recognized as an emerging infection [7].

This study aims to comprehensively analyze the demographic profile, clinical presentations, and outcomes of patients with melioidosis treated at Government Medical College, Kozhikode, over eight years.

## Materials and methods

Study design

This was a retrospective study conducted over eight years (September 2004 to August 2012) at the Government Medical College, Kozhikode, Kerala, India. It included a telephonic follow-up.

Data collection and participants

The study included all inpatients from whom *B. pseudomallei* was isolated from blood, pus, or other body fluids. Using a standardized proforma, we reviewed patient records to collect data on age, gender, occupation, geographic origin, clinical details, and outcomes (including fever defervescence, recovery, or death).

Diagnostic methods and management

Diagnosis of melioidosis required a comprehensive, multi-pronged approach. Accordingly, blood, urine, respiratory secretions, synovial, peritoneal, and pericardial fluid, sputum, pus, and wound swabs were collected for culture based on each patient's specific clinical presentation. Because melioidosis is classified as a "category 3" pathogen by the Advisory Committee on Dangerous Pathogens, the laboratory was promptly notified whenever melioidosis was suspected.

Initial identification of the organism relied on colony morphology and standard biochemical tests. This was followed by confirmatory testing, which included microscopic demonstration of small, bipolar, gram-negative rods with the characteristic "safety pin" appearance. The gold standard for diagnosis remained the culture of *B. pseudomallei* from any clinical specimen. Isolation was reliably achieved using standard culture media such as blood agar, MacConkey agar, and brain heart infusion broths, while the selective Ashdown's agar was used to ensure reliable isolation from specimens with normal or contaminating flora [8].

It is important to note that this study was conducted from September 2004 to August 2012 and included only cases diagnosed by bacterial culture. Although molecular methods such as polymerase chain reaction (PCR) offer rapid results with high sensitivity and specificity, they were not the primary diagnostic tools during most of the study period; we could confirm the diagnosis by PCR in only a few cases. Matrix-assisted laser desorption/ionization time-of-flight mass spectrometry (MALDI-TOF MS) was not available during the study period.

Antibiotic susceptibility testing showed that all isolates were sensitive to ceftazidime. The isolates also exhibited susceptibility to ciprofloxacin, cotrimoxazole, tetracycline, and chloramphenicol, but showed consistent resistance to gentamycin, amikacin, and ampicillin. Resistance to amoxicillin-clavulanic acid was also observed. Based on these sensitivity patterns, all patients in this study were treated with a standardized regimen. The intensive phase consisted of Inj. ceftazidime 2 gm intravenously (IV every eight hours for two weeks). This was followed by the eradication phase, which included oral trimethoprim-sulfamethoxazole 320:1600 mg (plus folic acid 5 mg daily) and doxycycline 100 mg every 12 hours for three months [9].

Patients were closely monitored, with follow-up every month or more often throughout the entire treatment period until recovery. Finally, to assess long-term outcomes, we conducted a telephonic follow-up in 2012.

Statistical analysis

Statistical analysis was performed using SPSS version 16.0 (SPSS Inc., Chicago, IL). Continuous data were expressed as mean and standard deviation (SD), and categorical data were presented as frequencies and percentages (n, %). The primary analytical objectives were to characterize the cohort and identify factors associated with patient outcomes. To identify predictors of mortality, survivors (n = 23) were compared with non-survivors (n = 10; one patient lost to follow-up was excluded from this analysis). Associations between categorical variables and mortality were tested using the chi-square test for variables with expected cell counts ≥5; for variables with expected cell counts <5, Fisher's exact test was used. Differences in continuous variables between groups were assessed using the independent samples t-test. Given the established role of DM as a significant risk factor, a secondary analysis was performed comparing the clinical and demographic profiles of diabetic versus non-diabetic patients. A two-tailed p-value of < 0.05 was considered statistically significant.

## Results

Demographics and clinical characteristics

A total of 34 patients with culture-confirmed melioidosis were treated during the study period. The mean age was 43 years (±14.8), ranging from 18 to 78 years. There was a strong male predominance, with males constituting 28 (82.4%) of the cases. Geographically, all patients were from Kerala, mostly from the districts of Kozhikode (22, 64.7%), Malappuram (8, 23.5%), Kannur (2, 5.9%), and Kasargod (1, 2.9%). One patient who recently relocated to Kerala from Uttar Pradesh for employment was an exception.

The mean hospital stay was 20 days (±16), ranging from three to 83 days. Regarding the duration of fever, the median was 20.5 days, with an interquartile range of 23 days; the shortest duration was two days, while the longest was an extreme case of 270 days. This particular patient was a 78-year-old male with DM and a current smoking habit, who presented in a drowsy state. His clinical picture included left knee joint arthritis, and the organism was isolated from both the joint aspirate pus and his blood. Table 1 summarizes the comprehensive demographic, clinical, and laboratory profile of the study cohort.

**Table 1:** Baseline characteristics of 34 patients with culture-proven melioidosis. SD, standard deviation; TLC, total leukocyte count; ESR, erythrocyte sedimentation rate.

Characteristic	Value
Age, years	
Mean ± SD	43.4 ± 14.8
Range	18 – 78
Gender, n (%)	
Male	28 (82.4)
Female	6 (17.6)
Major comorbidities, n (%)	
Diabetes mellitus	24 (70.6)
Hypertension	22 (64.7)
Chronic kidney disease	3 (8.8)
Alcohol use	7 (20.6)
Current smoking	9 (26.5)
Past history of tuberculosis	1 (2.9)
Common clinical features, n (%)	
Fever	30 (88.2)
Cough	17 (50.0)
Jaundice	8 (23.5)
Laboratory parameters	
TLC, cells/mm³	11,229 ± 5,090
ESR, mm/hr	96.1 ± 29.2
Platelet count, lakhs/μL	2.6 ± 1.2

Clinical outcomes

Regarding patient outcomes, 23 (67.6%) patients survived to discharge, 10 (29.4%) died during hospitalization, and one (2.9%) discontinued treatment and was lost to follow-up. A comparative analysis was conducted to identify factors associated with in-hospital mortality (23 survivors vs. 10 non-survivors). As shown in Table 2, non-survivors were older (mean age = 47.2 vs. 41.0 years) and had higher rates of DM (9 (90.0%) vs. 15 (65.2%)) and jaundice (4 (40.0%) vs. 4 (17.4%)). Septic arthritis was more common among survivors (10 (43.5%) vs. 1 (10.0%)). However, none of these associations reached statistical significance (p > 0.05).

**Table 2:** Comparison of clinical characteristics between survivors and non-survivors. Statistical comparisons: Chi-square or Fisher’s exact test for categorical variables; independent t-test for continuous variables. * Fisher's exact test was used due to small expected cell counts; χ² approximation values are provided for reference. SD, standard deviation; TLC, total leukocyte count; ESR, erythrocyte sedimentation rate.

Characteristic	Survivors (n = 23)	Non-survivors (n = 10)	Test statistic	p-value
Age, years (mean ± SD)	41.0 ± 13.8	47.2 ± 16.4	t = 1.12	0.256
Male, n (%)	20 (87.0)	8 (80.0)	χ² = 0.28	0.628
Diabetes mellitus, n (%)	15 (65.2)	9 (90.0)	χ² = 2.35	0.214
Hypertension, n (%)	16 (69.6)	6 (60.0)	χ² = 0.31	0.704
Jaundice, n (%)	4 (17.4)	4 (40.0)	χ² = 1.80*	0.214*
Septic arthritis, n (%)	10 (43.5)	1 (10.0)	χ² = 3.86*	0.113*
Positive blood culture, n (%)	13 (56.5)	6 (60.0)	χ² = 0.03	1.000
TLC >11,000/mm³, n (%)	9 (39.1)	6 (60.0)	χ² = 1.16*	0.285*
ESR, mm/hr (mean ± SD)	94.6 ± 28.8	99.1 ± 30.8	t = 0.42	0.681

Fever was the most common presenting symptom, occurring in 30 (88.2%) patients. It was characterized as high-grade intermittent in 17 (50.0%) and continuous in 11 (32.4%) patients. A wide range of other symptoms and signs was also observed, including rigors (7, 20.6%), headache (5, 14.7%), nausea and vomiting (4, 11.8%), and pustular skin lesions (1, 2.9%). Common clinical signs included pallor (7, 20.6%), jaundice (8, 23.5%), weight loss (7, 20.6%), and lymphadenopathy (7, 20.6%). Arthritis was present in nine (26.5%) patients. Cutaneous manifestations were observed in seven (20.6%) patients and consisted primarily of abscesses. Importantly, the organism was isolated from the blood in 19 (55.9%) subjects, indicating a high rate of bacteremia.

Diabetes as a comorbidity

Comorbidities were highly prevalent in our cohort, with 27 (79.4%) patients having at least one. DM was the most frequent, present in 24 (70.6%) patients. A focused analysis comparing patients with and without DM revealed significant differences: patients with DM were significantly older than those without DM (48.0 ± 13.7 years vs. 33.1 ± 9.8 years; p = 0.002) and had a higher prevalence of hypertension (19 (79.2%) vs. 3 (30.0%); p = 0.011). These findings are detailed in Table 3. Other notable comorbidities included alcohol dependence in seven (20.6%), chronic kidney disease in two (5.9%), of whom one patient was on hemodialysis, HIV infection in one (2.9%), post-splenectomy status in one (2.9%), and a previous history of tuberculosis in eight (23.5%).

**Table 3:** Comparison of characteristics between patients with and without diabetes mellitus. Statistical comparisons: Independent t-test for continuous variables; Chi-square or Fisher’s exact test for categorical variables. * Fisher's exact test was used due to small expected cell counts; χ² approximation values are provided for reference. Bold indicates statistical significance (p < 0.05). DM, diabetes mellitus; SD, standard deviation; TLC, total leukocyte count; ESR, erythrocyte sedimentation rate.

Characteristic	Patients with DM (n = 24)	Patients without DM (n = 10)	Test statistic	p-value
Age, years (mean ± SD)	48.0 ± 13.7	33.1 ± 9.8	t = 3.37	0.002
Male, n (%)	21 (87.5)	7 (70.0)	χ² = 1.48	0.326
Hypertension, n (%)	19 (79.2)	3 (30.0)	χ² = 8.04*	0.011*
Mortality, n (%)	9 (37.5)	1 (10.0)	χ² = 2.47*	0.214*
Septic arthritis, n (%)	7 (29.2)	4 (40.0)	χ² = 0.36*	0.694*
Positive blood culture, n (%)	13 (54.2)	6 (60.0)	χ² = 0.10	1.000
TLC, cells/mm³ (mean ± SD)	11,429 ± 5,440	10,730 ± 4,295	t = 0.38	0.706
ESR, mm/hr (mean ± SD)	98.6 ± 29.3	89.9 ± 29.0	t = 0.81	0.425

Spectrum of infection

The clinical spectrum of melioidosis revealed distinct patterns of organ system involvement. Analysis of infection burden revealed that five (14.7%) patients had a single focal site of infection, while 26 (76.5%) presented with multi-focal involvement (two or more organ systems). Musculoskeletal manifestations predominated, affecting 12 (35.3%) patients, with septic arthritis representing the most common focal infection (11 (32.4%) patients). This musculoskeletal preponderance contrasted with visceral involvement, which affected nine (26.5%) patients and demonstrated a notable pattern of hepatosplenic tropism. Liver and spleen were equally affected (five patients each), with two patients presenting concurrent hepatic and splenic abscesses, suggesting hematogenous dissemination.

Cutaneous manifestations exhibited a different clinical profile, with skin and soft tissue abscesses occurring in seven (20.6%) patients. The distribution of these abscesses favored the chest wall (three patients) and scalp (two patients), while paraspinal and retroperitoneal locations indicated more severe, deep-seated infections. Lymphatic involvement, observed in seven (20.6%) patients, was predominantly cervical, suggesting regional lymphatic spread from primary foci.

Pleuropulmonary disease represented a significant disease burden, affecting eight (23.5%) patients. This included both pleural space pathology (seven patients with empyema or effusion) and parenchymal disease (four patients with pneumonia or lung abscess), with some patients exhibiting both, indicating contiguous spread or severe systemic infection. The pattern of organ involvement suggested a hierarchy of vulnerability, with musculoskeletal > pleuropulmonary > cutaneous/lymphatic > visceral systems being most commonly affected.

**Figure 1:**
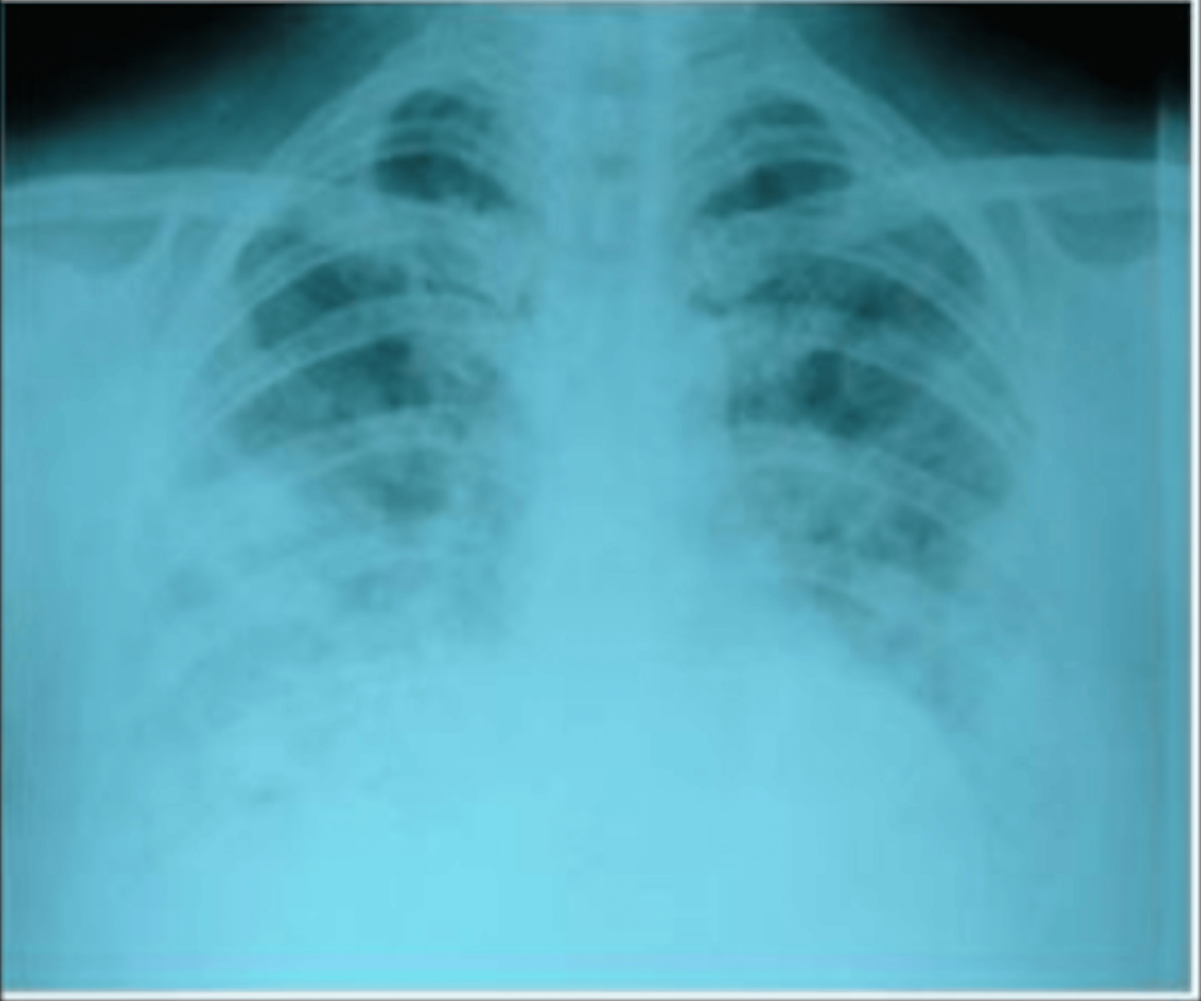
X-ray of the chest (posteroanterior view) showing non-homogeneous opacities in both mid and lower zones, more on the right side. Posteroanterior chest radiograph showing non-homogeneous opacities in both lung fields, consistent with bilateral pulmonary infiltrates.

**Figure 2:**
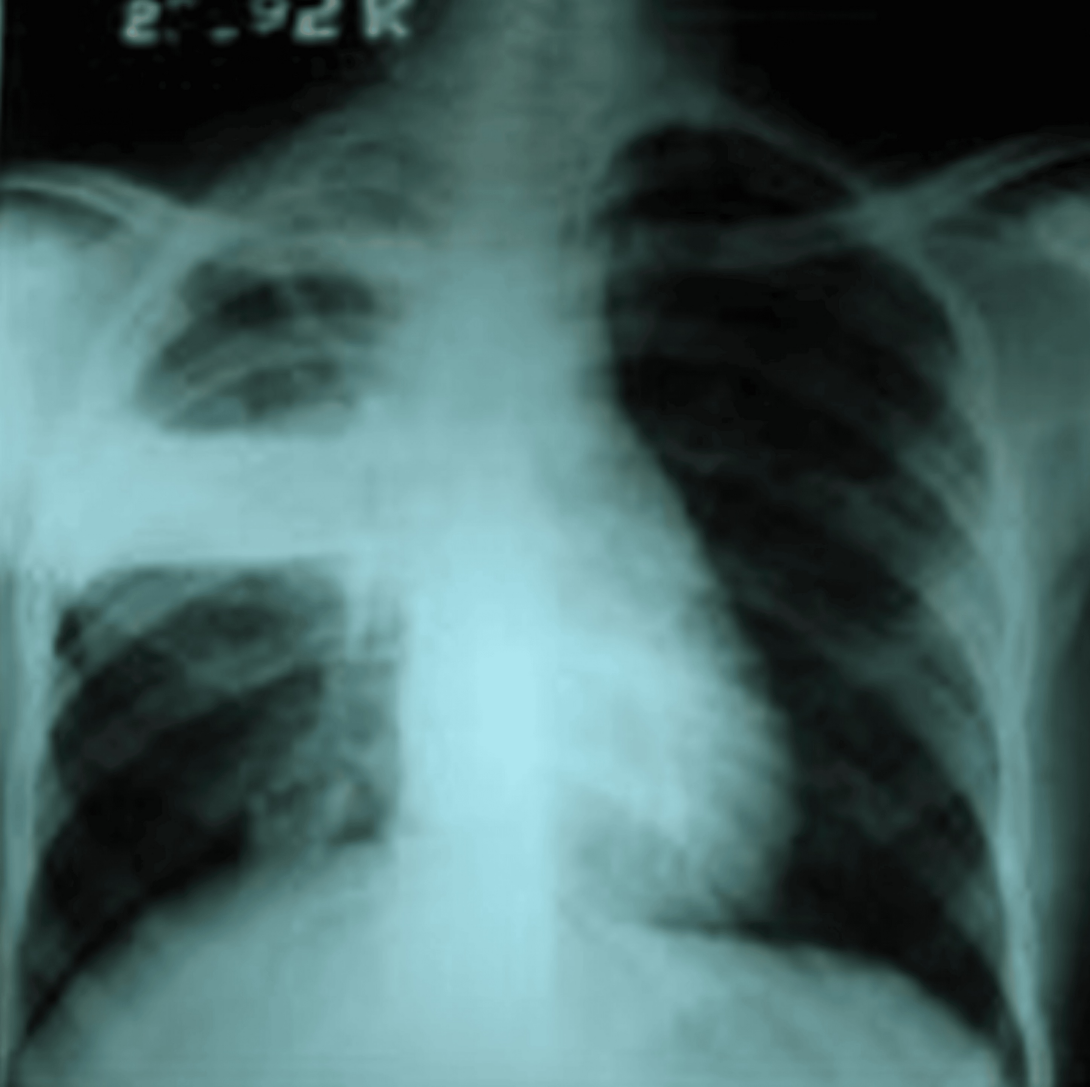
Chest radiograph showing right upper lobe cavitary lesion. Posteroanterior chest X-ray demonstrating a well-defined cavity with an internal air-fluid level in the right upper lobe, without associated fibrotic changes.

**Figure 3:**
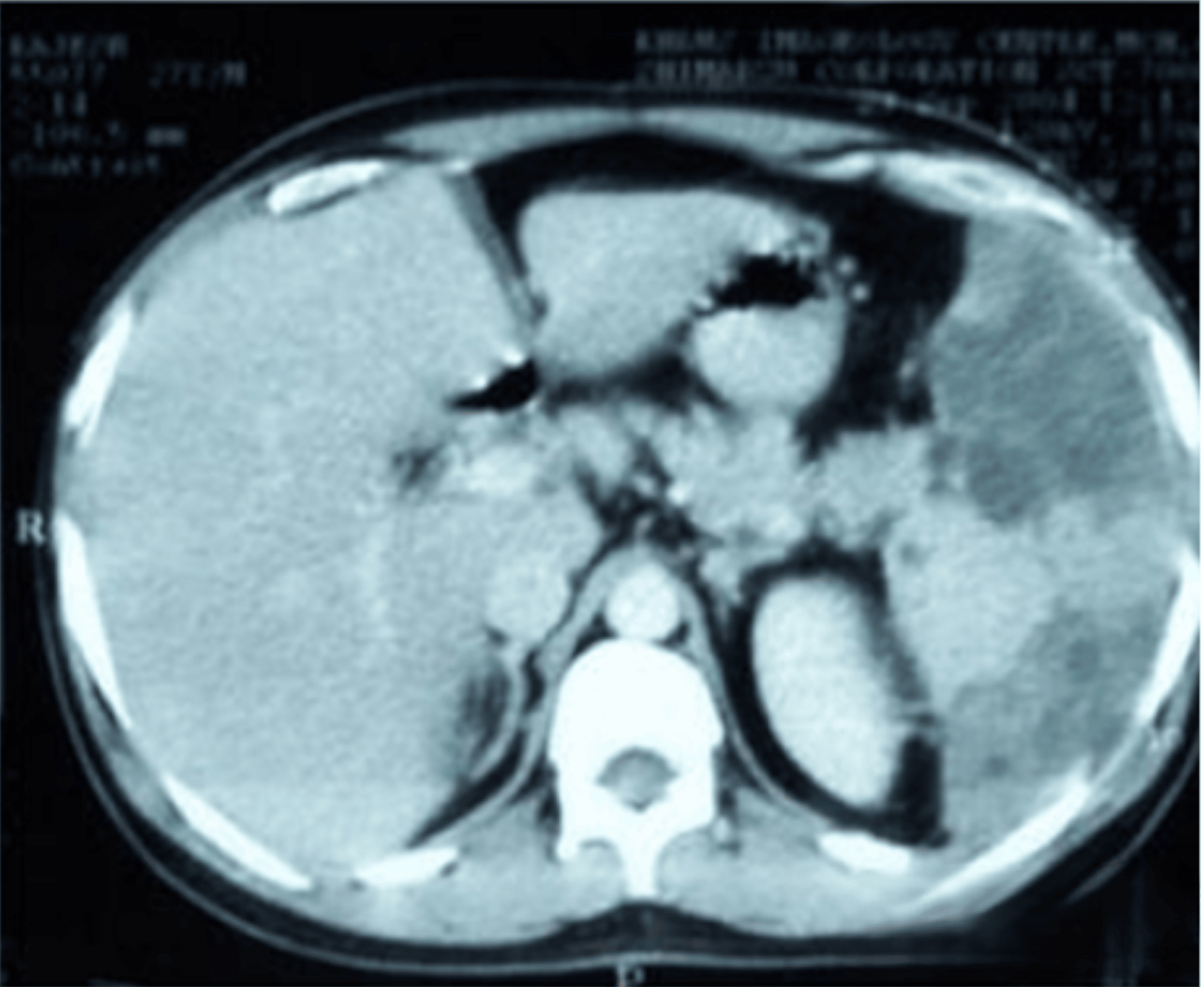
CT of the abdomen showing multiple hepatic and splenic abscesses. Axial contrast-enhanced CT scan of the abdomen demonstrating multiple, well-circumscribed hypodense lesions of varying sizes in both the liver and spleen, consistent with multiple visceral abscesses.

Comparative analysis showed that patients with musculoskeletal involvement generally had better outcomes (as indicated by Table 2, which reports a higher prevalence of septic arthritis among survivors). In contrast, visceral abscesses and pleuropulmonary disease were linked to more severe systemic illness. The specific locations of infections and abscesses, highlighting this hierarchical distribution and anatomical preference, are detailed in Table 4. Representative radiographic findings illustrating these patterns are shown in Figures 1-3.

**Table 4:** Systemic involvement and sites of infection (N = 34). A single patient could have involvement at multiple sites or systems; percentages are based on the total cohort (N = 34). MODS, multiple organ dysfunction syndrome.

Type of involvement	n (%)	Specific site	n
	12 (35.3)	Septic arthritis	11
Musculoskeletal		Osteomyelitis	1
Skin & soft tissue	7 (20.6)	Chest wall abscess	3
		Scalp abscess	2
		Paraspinal abscess	1
		Retroperitoneal abscess	1
		Neck abscess	1
Lymphatic	7 (20.6)	Cervical lymphadenopathy	5
Pleuropulmonary	8 (23.5)	Empyema/pleural effusion	7
		Lung abscess/pneumonia	4
Visceral	9 (26.5)	Liver abscess	5
		Splenic abscess	5
		Both liver & spleen	2
Other	2 (5.9)	Sepsis with MODS	2
	1 (2.9)	Genitourinary infection	1

Laboratory findings

Laboratory investigations offered further insights into the disease's profile. The mean hemoglobin level was 11 gm% (±2.5), with values ranging from 6.5 to 16.8 gm%. Markers of inflammation were notably elevated, as shown by a mean erythrocyte sedimentation rate (ESR) of 96 mm (±29) within the first hour, varying from 25 to 146 mm. Regarding leukocyte counts, no patients had a count below 4000/mm³, and 15 (44.1%) exhibited counts above 11,000/mm³. The differential leukocyte count consistently indicated a predominance of polymorphonuclear leukocytes. Finally, the Mantoux test was positive in two (5.9%) and negative in 11 (32.4%) of the patients tested.

Follow-up outcomes

Reiterating the outcomes for clarity, 23 (67.6%) patients survived and were discharged, while 10 (29.4%) died during the hospital stay. One patient (2.9%) was documented as having absconded after insisting on returning to his hometown despite being critically ill. During the telephonic follow-up, we found that two patients among the survivors had been readmitted with a relapse of the disease.

## Discussion

Our eight-year study provides a detailed clinical profile of melioidosis in a South Indian tertiary care center, confirming its status as a significant cause of morbidity and mortality. This analysis aligns with global data, showing that melioidosis, caused by the soil bacterium *B. pseudomallei*, is an emerging health threat in many tropical regions, including India [1,2]. While anyone can be infected, the disease disproportionately affects individuals with specific risk factors. As evidenced in our cohort and the wider literature [3], conditions like DM, excessive alcohol use, and chronic kidney disease significantly increase susceptibility and mortality.

The diagnostic process for melioidosis presents numerous challenges. First, its classification as a "category 3" pathogen requires strict laboratory safety measures. Second, its varied symptoms, as "the great mimicker," call for a high level of clinical suspicion. In our setting, diagnosis depended on a combination of clinical evaluation and laboratory confirmation. While colony morphology, biochemical testing, and microscopic identification of characteristic "safety pin" bacilli were essential, culture remained the gold standard [8]. Although advanced molecular techniques such as PCR provide faster results and specificity, they were not widely accessible during our study period, highlighting the changing landscape of melioidosis diagnostics.

The demographic patterns we observed align closely with other studies [10,11]. The notable male predominance likely reflects increased occupational exposure to soil and water in this region. Similarly, the wide age range (18-78 years) and an average age of 43 confirm that melioidosis can affect working-age adults across all ages. Additionally, our statistical analysis identified a distinct clinical phenotype among affected individuals: patients with DM were significantly older than their non-diabetic counterparts and had a much higher prevalence of hypertension. This pattern indicates that, within our endemic region, melioidosis tends to affect middle-aged adults with a cluster of metabolic comorbidities. Geographically, the concentration of cases in Kozhikode and Malappuram districts highlights these areas as endemic within Kerala, but further multicentric studies are needed to confirm this, as these districts are where the majority of patients report to this tertiary care center. This finding should inform public health surveillance efforts. Overall, these results enhance understanding of the demographic patterns and clinical features of melioidosis in our study population, supporting the development of targeted interventions and management strategies for this infectious disease [12].

The clinical management of melioidosis is notably complex [13], as reflected in the prolonged and variable hospital stays (3-83 days) and the extended duration of fever in our patients. Treatment of melioidosis remains a significant challenge, even in settings with adequate resources to support patients with multiple organ failure and extensive clinical experience. A critical therapeutic pitfall is that antibiotics commonly used for first-line treatment of gram-negative septicemia (e.g., ampi-/amoxicillin, aminoglycosides, and ceftriaxone) are ineffective. This fact highlights the importance of clinical suspicion and early recognition of melioidosis when starting presumptive antimicrobial therapy. Therefore, early diagnosis and prompt initiation of appropriate treatment, such as ceftazidime, are essential. The relapses observed in two of our survivors further illustrate the persistent nature of this infection and the need to complete the full eradication therapy.

Our outcomes emphasize the severity of the disease. The survival rate of 23 (67.6%) demonstrates the effectiveness of the recommended antibiotic protocol when followed. However, the high mortality rate of 10 (29.4%) serves as a stark reminder of the lethal potential of melioidosis, especially when diagnosis or treatment is delayed [14]. A comparison of survivors and non-survivors revealed trends toward higher mortality among older patients (47.2 vs. 41.0 years), those with DM (9 (90.0%) vs. 15 (65.2%)), and those with jaundice (4 (40.0%) vs. 4 (17.4%)). However, none of these associations reached statistical significance in our cohort, likely reflecting the limited sample size and number of mortality events. Notably, septic arthritis was more common among survivors than non-survivors (10 (43.5%) vs. 1 (10.0%)), suggesting that localized joint involvement may correlate with a less severe disease course compared to disseminated infection with bacteremia. This finding, while requiring confirmation in larger studies, aligns with the understanding that focal infections often indicate better outcomes than systemic sepsis. The predominant presentation was fever, along with a wide range of focal symptoms across multiple organ systems, which powerfully illustrates the systemic and diverse nature of this infection [4,15]. The complexity is further highlighted by the presence of conditions such as lymphadenopathy and multi-site skin infections, which require comprehensive management strategies.

From a laboratory perspective, the frequent anemia and markedly elevated inflammatory markers, such as ESR, reflect the significant systemic inflammatory burden of melioidosis [16,17]. The leukocyte response, with no cases of leukopenia and a notable proportion of patients (15, 44.1%) exhibiting leukocytosis with polymorphonuclear predominance, is characteristic of an acute bacterial infection. Interestingly, routine inflammatory markers, specifically total leukocyte count and ESR, did not differ significantly between survivors and non-survivors (p > 0.05). This indicates that while these parameters are elevated in systemic inflammation, they may lack specific prognostic value in predicting clinical outcomes in melioidosis, a limitation consistent with observations in other severe infections [4]. Collectively, these findings underscore the need for more specific and reliable biomarkers to improve risk stratification and guide clinical management in melioidosis [18].

The predominance of DM as a comorbidity, affecting 24 (70.6%) patients in our cohort, is a central finding of our study and aligns with meta-analytic evidence demonstrating a three-fold increased risk of melioidosis in populations with DM [19]. More importantly, our comparative analysis revealed that while DM identifies the susceptible host, it did not significantly alter the disease's core clinical features, namely, the prevalence of key symptoms, focal infections, and inflammatory markers was similar between patients with and without DM. This highlights a crucial clinical point: because the risk is much higher and the presentation is non-specific and similar in patients without DM, melioidosis must be considered in the differential diagnosis of any patient with DM presenting with an acute febrile illness, sepsis, or focal infection, such as pneumonia or abscesses.

A major diagnostic challenge in our endemic region is differentiating melioidosis from tuberculosis. Acute pulmonary melioidosis can present as diffuse nodular infiltrates that coalesce, cavitate, and often progress rapidly. Upper lobe changes with infiltrates and/or cavitation can be seen on chest imaging in chronic pulmonary melioidosis, which can mimic pulmonary tuberculosis [20]. This diagnostic dilemma is compounded by the fact that eight (23.5%) patients in our cohort had a previous history of tuberculosis, creating a clinical background where both infections need to be considered. This is further emphasized by the finding that the Mantoux test was positive in only two (15.4%) of 13 patients tested (15.4%), despite the clinical and histopathological similarities between the two diseases [3,21]. Therefore, melioidosis should be regarded as an important differential diagnosis not only in patients suspected of having active tuberculosis, but also in those with a history of tuberculosis who present with compatible symptoms, especially when there is a poor response to anti-tubercular therapy or repeated negative acid-fast bacilli results. This diagnostic overlap underscores the importance of heightened clinical suspicion and appropriate microbiological testing for melioidosis in regions where both infections are common.

This study has certain limitations that should be recognized. The single-center, retrospective design and the relatively small sample size are inherent limitations that influence the statistical power of our analyses and the generalizability of the results. Furthermore, the extended study period means that diagnostic technologies have evolved, which we addressed by relying on the consistent gold standard of culture for case inclusion. Lastly, it is essential to note that data collection ended in 2012; therefore, the clinical landscape, including management protocols and local epidemiology, may have changed in the intervening years. Despite these points, the study offers a solid, culture-confirmed baseline description of the clinical spectrum of melioidosis in this region, which remains crucial for understanding the disease's presentation and severity.

However, these clinical and laboratory findings enhance a comprehensive understanding of melioidosis, thus supporting more timely diagnosis, effective monitoring, and better management of this complex infectious disease.

## Conclusions

This study emphasizes melioidosis as a serious and endemic public health threat in this part of India, associated with high mortality rates and a strong correlation with DM. Patients with diabetes who had melioidosis were usually older and more often had hypertension. The diverse and often non-specific clinical presentations necessitate a high index of suspicion, especially in individuals with diabetes or occupational exposure to soil and water.

Our experience confirms that outcomes can be positively influenced by the consistent application of a two-phase antibiotic protocol, starting with intravenous ceftazidime. Therefore, the cornerstone of management remains early clinical recognition, prompt diagnostic sampling for culture, and immediate initiation of appropriate antimicrobial therapy. Future efforts should focus on increasing physician awareness and enhancing laboratory surveillance to enable timely diagnosis and improve overall survival rates.

## Appendices

Glossary

(1) Human pathogen hazard group 3: Can cause severe human disease and may be a serious hazard to employees; it may spread to the community, but there is usually effective prophylaxis or treatment available.

(2) Bioterrorism agent: Bioterrorism involves intentionally releasing an agent like viruses, bacteria, or toxins to harm people, livestock, or crops.
